# Editorial for the Special Issue “Sensing Brain Activity Using EEG and Machine Learning”

**DOI:** 10.3390/s24082535

**Published:** 2024-04-15

**Authors:** Peter Rogelj

**Affiliations:** Faculty of Mathematics, Natural Sciences and Information Technologies, University of Primorska, Glagoljaška 8, 6000 Koper, Slovenia; peter.rogelj@upr.si

Sensing brain activity to reveal, analyze and recognize brain activity patterns has become a topic of great interest and ongoing research. One of the most popular methods to obtain data from brain activity is electroencephalography (EEG); however, the data need to be processed and analyzed to reveal meaningful insights. The field of EEG analysis is undergoing rapid development and transformation by utilizing modern machine learning techniques. The objectives of its applications are diverse, from revealing the complexity of brain functioning to paving the way for seamless human–computer interfaces. This Special Issue explores the current directions and trends of EEG analysis, highlighting the shift from conventional statistical methodologies to the application of modern machine learning approaches. It includes seven original research articles that have successfully completed a rigorous peer-reviewing process.

There are two main streams of EEG analysis. The first one is an investigatory analysis, which tends to support the study of brain functioning and has the ability to reveal mental states and neurological disorders. Neurological research and medical diagnosis are its main uses. The conventional approaches applied here extract features from EEG signals in the temporal, spatial, and/or frequency domains, which provide human-understandable information describing the neurological background of brain functioning. The second stream is classificatory analysis, wherein the comprehension of neurological backgrounds is not necessary, and only the accomplishment of specific application objectives is considered. This stream is exemplified by Brain–Computer Interfaces (BCIs).

Both of the streams need to distinguish, learn, and recognize complex EEG signal patterns. The classificatory one, due to its nature, relies on machine learning techniques, while the investigatory analysis is increasingly using it as a replacement for statistical analyses. The benefits of machine learning include the ability to discover more complex patterns with higher prediction accuracy. Machine learning has been revolutionized by the introduction of artificial neural networks, specifically deep learning. In other areas of data analysis, such as computer vision, deep learning has already proven its capabilities, providing higher accuracy, reliability, and adaptability than conventional machine learning methods. However, this paradigm shift is not without its challenges, and the scientific community actively engages with the ongoing problems in EEG analysis. Specifically, deep learning models require extensive training on large datasets, and do not directly provide an explanation of the decision-making process.

Some articles in this Special Issue seek an optimal balance between conventional and neural network approaches for specific subdomains of EEG analysis. Batistic et al. (contribution 1) compared several classifiers for the classification of motor imagery with and without vibrotactile guidance, and concluded that neural network-based classifiers outperform conventional ones, while vibrotactile guidance contributes more to conventional ones. Mortier et al. (contribution 2) analyzed human attention and the influences of auditory and visual support on recognizing attention using EEGs. The classification was performed using four different machine learning methods. The best-performing one was the end-to-end neural network EEGnet. The authors have demonstrated the explainability of such neural network models’ decisions using saliency maps. Xia et al. (contribution 3) proposed an approach to emotion classification based on a bidirectional long short-term memory neural network. They introduced a novel individual difference module (IDM) that adapts the general model to individuals, thereby resolving a significant concern regarding intersubject variability, which is crucial for various EEG signal classification tasks. Mocilnik et al. (contribution 4) proposed an approach to classifier modeling for a limited amount of data. The goal was to distinguish pre- and post-rehabilitation states of Broca’s aphasia. The method combines both conventional feature extraction using connectivity metrics and a neural network classifier.

Other articles in this Special Issue employ conventional classifiers and optimize feature extraction and analysis for specific tasks. Mumenin et al. (contribution 5) focused on the detection of seizures in neonates. They proposed an optimization of ML hyperparameters to obtain a higher final accuracy. The accuracy obtained is higher than for the other conventional approaches to which the authors compare their proposed approach, but lower than the ones obtained using deep learning approaches. However, this could help direct further research in EEG ML, as deep learning hyperparameters also highly affect models’ accuracy, especially when the amount of data are limited. Ozawa et al. (contribution 6) presented a complex experiment to detect congruence between visual and tactile stimuli. The extra complexity is due to the analysis of how the material stimulation modulates an electrically stimulated SEP. They managed to detect congruence with accuracies significantly higher than the baseline. The analysis yields additional information about brain regions involved in such discrimination, contributing to the understanding of sensory processing in the brain. Wang et al. (contribution 7) demonstrated the concept of using state-space model parameters for EEG classification. The advantage was shown in subject-independent modeling for detecting seizures, where the proposed model performs better than the compared methods.

This Special Issue captures the pulse of EEG research, bringing to light the challenges, breakthroughs, and future prospects. The convergence of EEG and neural networks heralds a new era where the intricate dance of brainwaves meets the precision of artificial intelligence, unlocking numerous possibilities for scientific exploration and technological innovation.

